# Comparison of Clinical and Radiological Findings for the Prediction of Scar Integrity in Women With Previous Lower Segment Cesarean Sections

**DOI:** 10.7759/cureus.43976

**Published:** 2023-08-23

**Authors:** Pooja Patil, Nishi Mitra, Smita Batni, Megha Jain, Shesha Sinha

**Affiliations:** 1 Obstetrics and Gynaecology, LN Medical College and Research Center, Bhopal, IND; 2 Radiology, LN Medical College and Research Center, Bhopal, IND

**Keywords:** elective lscs, lscs, lower segment caesarean section, cesarean section, scar tenderness, scar dehiscence, scar rupture, scar integrity, scar thickness, previous cesarean section

## Abstract

Introduction: We aimed to compare the clinical and radiological findings to predict scar integrity in term antenatal mothers with a previous lower segment cesarean section (LSCS).

Methodology: This prospective study was conducted in the obstetrics and gynecology department of LN Medical College, Bhopal, India, from August 2020 to August 2021. We included all pregnant women with term gestation (37+0 to 42+0 weeks) who were admitted either for elective repeat LSCS or for emergency LSCS and had a history of a previous LSCS. A detailed history and clinical examinations were performed. We noted the presence of scar tenderness and conducted transabdominal ultrasound (USG) to assess the integrity of the uterine scar in all women. During surgery, the surgeon identified the lower uterine segment scar and graded it as normal, thinned-out, dehiscent, or ruptured. We calculated sensitivity, specificity, positive predictive value (PPV), and negative predictive value (NPV) for both clinical findings (scar tenderness) and ultrasound findings as predictors of scar integrity.

Results: A total of 60 pregnant women were included in the study. During a repeat cesarean section, we found a thinned-out scar in 26 women out of 60 (43.3%). Out of 60 women, 13 had scar tenderness, and among these 13 women, 12 had thinned-out scars intraoperatively. Forty-seven women had no scar tenderness; 14 had thinned-out scars intraoperatively. The sensitivity of scar tenderness as a predictor of a thinned-out scar was 46.2%, specificity was 97.1%, PPV was 92.3%, and NPV was 70.2%. Whereas the sensitivity of ultrasound scar thickness as a predictor of a thinned-out scar was only 19.2%, with a specificity of 94.1%, a PPV of 71.4%, and an NPV of 60.4%. Thus, we documented a significant correlation between intraoperative and clinical findings (κ = 0.46; p<0.05), but no agreement could be found between ultrasound and intraoperative findings (p>0.05).

Conclusions: Clinically evident scar tenderness continues to be a useful parameter to predict intraoperative scar status.

## Introduction

Lower segment cesarean section (LSCS) is one of the most common surgical procedures performed in obstetrics. Globally, the rate of cesarean sections varies from 10% to 25%, and this rate is showing an increasing trend [1]. In India, about 47.4% of births in private and 14.3% of births in public hospitals were via LSCS, according to National Family Health Survey (NFHS-5) 2019-21 data. This was higher as compared to NFHS-4 2015-16 data, which showed 40.9% of births in private and 11.9% of births in public institutions via LSCS [2]. The most common indication of a cesarean section is the previous cesarean section, which accounts for one-third of all cesarean sections. Thus, decreasing the elective repeat cesarean section may decrease the overall rate of cesarean births [3].

Trial of labor after a previous cesarean section or vaginal birth after cesarean (VBAC) remains a dilemmatic issue as there is no consensus regarding when to proceed for vaginal delivery or when to do a repeat cesarean section [3]. The morbidity associated with a trial of VBAC is an area of concern, as the dreaded complication of vaginal birth after a previous cesarean section is uterine rupture [4]. The risk of uterine rupture in previous cesarean section cases undergoing VBAC is 0.9% [5].

Various factors affect the outcome of vaginal birth after a cesarean section, such as the time interval between the previous cesarean section and the current pregnancy, previous successful vaginal deliveries, indications of a cesarean section previously, the presence of wound sepsis, and others. None of the obstetricians are in favor of facing medicolegal issues in case of any mishaps; therefore, there is a tendency to avoid trying for a VBAC.

Ultrasound can be used to assess the thickness as well as the integrity of the scar of the lower uterine segment to estimate the risk of uterine rupture or scar dehiscence during the labor trial [6,7]. But its use is not conclusive and lacks efficient evidence for its continued use in the assessment of scar integrity. The complications that may occur at the time of delivery may be recognized early at the time of antenatal visits. However, a significant majority of women in developing countries usually attend less than four antenatal visits or attend the antenatal clinic late during the second or third trimester. Women may present in labor for the first time with the history of previous cesarean sections, wherein deciding the mode of delivery is very difficult [8,9,10].

With the above background, the study aimed to compare the clinical findings of scar tenderness and radiological findings of scar thickness and to assess their correlation with the intraoperative lower uterine segment scar status in term antenatal women with previous cesarean sections.

## Materials and methods

This study was conducted as a prospective study in the obstetrics and gynecology department of LN Medical College, Bhopal, India, after obtaining approval from the Institutional Ethics Committee of LN Medical College and JK Hospital (Reference Letter No. LNMC&Rc/Dean/2020/Ethics/145). The study was conducted from August 2020 to August 2021, during which all the pregnant women presenting with term gestation (37+0 to 42+0 weeks) with a history of a previous cesarean section who gave consent for participation in the study were included. However, women with placenta previa, a history of surgery on the uterus for other reasons like myomectomy, uterine anomalies, and previous LSCS who delivered vaginally were excluded from the study. Written and informed consent was obtained from all participating women.

Detailed history regarding sociodemographic variables, along with presenting complaints, obstetric history, history of previous cesarean delivery, its indication, intrapartum, and postpartum complications, if any, were noted. All the women were subjected to detailed abdominal and obstetric examinations, and findings were noted. An abdominal examination was also done to assess the scar tenderness. Transabdominal ultrasonography using a curvilinear probe was done in the full bladder state at term gestation or before elective repeat cesarean sections to assess the scar thickness. We ensured that the ultrasonography was done single-handedly by an experienced radiologist at our institute. A scar thickness of less than 2 mm was considered a strong positive predictor for scar dehiscence [11].

During surgery, the lower uterine segment scar was identified by the surgeon, and the scar was graded as normal, thinned-out, dehiscent, or ruptured. The thickness of the lower uterine segment (LUS) was categorized intraoperatively into four grades [12], which were:

Grade 1: a well-formed lower uterine segment [12]; Grade 2: a thin uterine scar but no uterine contents are visible. [12]; Grade 3: scar dehiscence as partial-thickness myometrial loss of integrity [12]; Grade 4: a ruptured scar with a full-thickness separation of the uterine wall and overlying serosa [12]

The statistical analysis was done using IBM SPSS software, version 20 (IBM Corp., Armonk, NY, USA). Data were presented either as mean and standard deviation, or frequency and percentage. The clinical-radiological correlation was done using the Chi-square test. Kappa statistics were applied to assess the level of agreement between intraoperative and clinical-radiological findings. A p-value of less than 0.05 was considered statistically significant.

## Results

The mean age of women was 28.67±4.75 years, and most of them belonged to the age range of 26 to 30 years (41.7%). About 93.3% of women were booked, and all were in term gestation. The mean duration between the previous and current LSCS was 4.02 ± 2.9 years (Table 1).

**Table 1:** Distribution of participants according to baseline variables

Baseline variables		Frequency (n=60)	Percentage (%)
Age (years)	20-25	19	31.7
	26-30	25	41.7
	31-35	11	18.3
	>35	5	8.3
Booking status	Booked	56	93.3
	Unbooked	4	6.7
Gestation period	37 -38 weeks	29	48.3
	38-39 weeks	25	41.7
	>39 weeks	6	10.0
Gravida	2	38	63.3
	3	16	26.7
	4	4	6.7
	5	1	1.7
	6	1	1.7
Parity	1	54	90.0
	2	5	8.3
	3	1	1.7
Mean duration from the last lower segment cesarean section (years)		4.02 ± 2.9	

According to the ultrasound reports, the LUS scar thickness was </=2 mm in seven women; 53 women had a scar thickness > 2 mm out of a sample size of 60. The mean scar thickness on the ultrasounds was 3.4±1.4 mm (Table 2).

**Table 2:** Patient distribution according to ultrasonography (USG) findings

USG		Frequency (n=60)	Percentage (%)
Scar thickness (mm)	≤2	7	11.7
	2.1-4	35	58.3
	>4	18	30.0

During a repeat cesarean section, we found thinned-out scars in 26 women out of 60 (43.3%), and the rest had normal intraoperative scar findings (Table 3).

**Table 3:** Patient distribution according to the intraoperative scar status

		Frequency (n=60)	Percentage (%)
Intraoperative scar status	Normal	34	56.7
	Thinned-out	26	43.3

On clinical examination, out of 60 women, 13 had scar tenderness, and 47 had no scar tenderness. But among those 13 women, 12 had thinned-out scars intraoperatively, and out of 37 with no scar tenderness, 14 women had thinned-out scars. The sensitivity of scar tenderness as a predictor of a thinned-out scar was 46.2%, with a specificity of 97.1%, a positive predictive value (PPV) of 92.3%, and a negative predictive value (NPV) of 70.2% (Table 4).

**Table 4:** The correlation of scar tenderness with intraoperative scar status

Scar tenderness	Intraoperative scar status	
	Thinned-out	Normal
Present	12	1
Absent	14	33

On correlating the ultrasound findings with intraoperative scar status, a total of seven women had a scar thickness of </= 2 mm, and among them, five had thinned-out scars. Fifty-three women had a scar thickness > 2 mm, and among them, 21 women had an intraoperatively thinned-out scar (Table 5).

**Table 5:** The correlation of scar thickness determined on ultrasonography with intraoperative scar status

Scar thickness on USG	Intraoperative scar status	
	Thinned-out	Normal
<2 mm	5	2
>2 mm	21	32

Out of 26 thinned-out scars, the clinical examination could identify scar tenderness in 12 (46.2%) women, whereas ultrasound revealed scar thinning in only five out of 26 women. This shows that the sensitivity of scar tenderness as a predictor of a thinned-out scar was 46.2%, specificity was 97.1%, PPV was 92.3%, and NPV was 70.2%. Whereas the sensitivity of ultrasound scar thickness as a predictor of a thinned-out scar is only 19.2%, with a specificity of 94.1%, a PPV of 71.4%, and an NPV of 60.4%. Thus, we documented a significant correlation between intraoperative and clinical findings (κ=0.46; p<0.05), but no agreement could be found between ultrasound and intraoperative findings (p>0.05). The complications faced during repeat cesarean sections were bladder adhesion (6.7%), low-lying placenta (1.7%), postpartum hemorrhage (1.7%), and peritoneal adhesions (8.3%) (Tables 6-7).

**Table 6:** Correlation of intraoperative scar status with clinical examination (scar tenderness) and USG scar thickness PPV: positive predictive value; NPV: negative predictive value

USG and clinical examination		Intraoperative scar status		Sensitivity (%)	Specificity (%)	PPV (%)	NPV (%)	K-Value	P-Value
		Normal	Thinned out						
		N (%)	N (%)						
Scar tenderness	Absent	33 (97.1)	14 (53.8)	46.2	97.1	92.3	70.2	0.46	0.001
	Present	1 (2.9)	12 (46.2)						
USG scar thickness	<2 mm	2 (5.9)	5 (19.2)	19.2	94.1	71.4	60.4	0.15	0.11
	>2 mm	32 (94.1)	21 (80.8)						

**Table 7:** Complications during a repeat cesarean section

Complications		Frequency (n=60)	Percentage (%)
	Bladder adhesion	4	6.7
	Low-lying placenta	1	1.7
	Postpartum hemorrhage	1	1.7
	Peritoneal adhesions	5	8.3
	None	49	81.7

## Discussion

The uterus, especially the lower segment of the uterus, plays an important role in LSCS. Ultrasounds and clinical examinations are important components of routine antenatal care. It is through these examinations that impending complications that may occur during labor can be identified, and thus, the management strategy and mode of delivery can be planned early [13].

Ultrasonography is a first-line, non-invasive technique that allows the visualization of morphology, along with any other pathology of the uterus. It is also helpful in calculating fetal indices, fetal well-being, amniotic fluid status, and other necessary parameters. Ultrasonography can also be utilized for assessing the lower uterine segment scar thickness [14]. We conducted the ultrasound for assessment of the uterine scar at term gestation. Similarly, in several other studies, ultrasound was performed for scar thickness in the last trimester, as the lower uterine segment is well-formed during this period [15,16,17].

Transabdominal ultrasound was utilized to assess the scar thickness in our study. The mean scar thickness in women with previous cesarean sections in our study was 3.4±1.4 mm. The cut-off for scar thickness to be able to predict the risk of uterine rupture is variable; some researchers suggest a cut-off below 2 mm [18], whereas others have suggested a cut-off of 3.5 mm [19]. The literature suggests that the lower uterine segment scar can be visible in approximately one-third of the cases, and there is an inverse relationship between the risk of uterine rupture and the assessed scar thickness with the aid of sonography [16].

On clinical examination, scar tenderness could be detected in 13 (21.7%) women out of 60 women. However, Singh et al. observed scar tenderness in 8.9% of cases, due to which VBAC could not be done to avoid the risk of uterine rupture [3].

Intraoperatively, scar thickness was assessed by the operating surgeon, and scar thinning was noted in 43.3% of women in our study. We documented that clinical examination could identify at least 46.2% of the women, whereas ultrasonography was less helpful in identifying the risk of scar rupture as it could predict it in only five out of 26 women. Overall, in our study, the sensitivity and specificity of abdominal ultrasound for predicting thinned-out scars were 19.2% and 94.1%, respectively. Sen et al., on the other hand, reported sensitivity and specificity of 90.9% and 84%, respectively, at a cut-off of 2.5 mm [20]. In a meta-analysis done in 2013, the pooled sensitivity and specificity of myometrial lower uterine segment thickness for cut-offs between 0.6 and 2.0 mm were 0.76 (95% confidence interval (CI), 0.60-0.87) and 0.92 (95% CI, 0.82-0.97); cut-offs between 2.1 and 4.0 mm reached a sensitivity and specificity of 0.94 (95% CI, 0.81-0.98) and 0.64 (95% CI, 0.26-0.90) [14].

Though we documented no significant clinical-radiological correlation, the correlation between clinical examination (scar tenderness) and intraoperative scar status was found to be statistically significant (p<0.05). Our study findings were concordant with the findings of the study done by Gupta et al. [21] and Khalil et al. [22], in which a significant association between scar tenderness and intraoperative scar dehiscence was reported. Thus, clinical examination is more reliable for estimating the risk of uterine rupture. The use of the transabdominal modality of ultrasonography for the assessment of scar status was the major limitation in our study, as previous studies have documented the higher utility of transvaginal ultrasonography for the assessment of scar thickness [3,15]. Another limitation is our small sample size, which is attributable to the COVID-19 pandemic during the period of study.

## Conclusions

Our study highlighted the importance of clinical examination of the scar in term gestation, as the presence of scar tenderness was a significant predictor of a thinned-out intraoperative scar compared to ultrasonography. In our study, radiological cesarean scar thickness did not have a significant correlation with intraoperative scar status, recommending that clinically evident scar tenderness continues to be a useful parameter to predict intraoperative scar status. In the future, the radiological evaluation of scar morphology may have a bearing on intraoperative scar status, and therefore further studies can be done on it.
